# A Role of Supraspinal Galanin in Behavioural Hyperalgesia in the Rat

**DOI:** 10.1371/journal.pone.0113077

**Published:** 2014-11-18

**Authors:** Diana Amorim, Ana David-Pereira, Patrícia Marques, Sónia Puga, Patrícia Rebelo, Patrício Costa, Antti Pertovaara, Armando Almeida, Filipa Pinto-Ribeiro

**Affiliations:** 1 Life and Health Sciences Research Institute (ICVS), School of Health Sciences (ECS), University of Minho, Braga, Portugal; 2 ICVS/3B's - PT Government Associate Laboratory, Braga/Guimarães, Portugal; 3 Institute of Biomedicine/Physiology, University of Helsinki, Helsinki, Finland; University of California, Los Angeles, United States of America

## Abstract

**Introduction:**

In chronic pain disorders, galanin (GAL) is able to either facilitate or inhibit nociception in the spinal cord but the contribution of supraspinal galanin to pain signalling is mostly unknown. The dorsomedial nucleus of the hypothalamus (DMH) is rich in galanin receptors (GALR) and is involved in behavioural hyperalgesia. In this study, we evaluated the contribution of supraspinal GAL to behavioural hyperalgesia in experimental monoarthritis.

**Methods:**

In Wistar-Han males with a four week kaolin/carrageenan-induced monoarthritis (ARTH), paw-withdrawal latency (PWL) was assessed before and after DMH administration of exogenous GAL, a non-specific GALR antagonist (M40), a specific GALR1 agonist (M617) and a specific GALR2 antagonist (M871). Additionally, the analysis of c-Fos expression after GAL injection in the DMH was used to investigate the potential involvement of brainstem pain control centres. Finally, electrophysiological recordings were performed to evaluate whether pronociceptive On- or antinociceptive Off-like cells in the rostral ventromedial medulla (RVM) relay the effect of GAL.

**Results:**

Exogenous GAL in the DMH decreased PWL in ARTH and SHAM animals, an effect that was mimicked by a GALR1 agonist (M617). In SHAM animals, an unselective GALR antagonist (M40) increased PWL, while a GALR2 antagonist (M871) decreased PWL. M40 or M871 failed to influence PWL in ARTH animals. Exogenous GAL increased c-Fos expression in the RVM and dorsal raphe nucleus (DRN), with effects being more prominent in SHAM than ARTH animals. Exogenous GAL failed to influence activity of RVM On- or Off-like cells of SHAM and ARTH animals.

**Conclusions:**

Overall, exogenous GAL in the DMH had a pronociceptive effect that is mediated by GALR1 in healthy and arthritic animals and is associated with alterations of c-Fos expression in RVM and DRN that are serotonergic brainstem nuclei known to be involved in the regulation of pain.

## Introduction

Galanin (GAL) is an injury-responsive peptide that is dramatically upregulated in the dorsal root ganglia and spinal dorsal horn interneurones during inflammation [1] or after nerve injury [2]. In healthy animals, GAL's action on nociceptive processing in the spinal cord is bidirectional, with low concentrations eliciting pronociceptive actions [3] and high concentrations promoting antinociception [4]. Differences in spinal actions of GAL also vary with the differential availability/activation of GAL receptor (GALR) subtypes. GALR1 has an inhibitory action and is more abundant than GALR2 (excitatory) and GALR3 (inhibitory) in the superficial dorsal horn [5]. Despite the considerable number of works evaluating its action in the peripheral nervous system and at the spinal cord level, the role of GAL in pain modulation at the supraspinal level is mostly unknown.

In basal conditions several studies showed that, both in humans and rodents, GAL is expressed in the supraoptic nucleus, the paraventricular nucleus of the hypothalamus, the dorsomedial hypothalamic nucleus (DMH), the arcuate nuclei, the lateral hypothalamic area, the locus coeruleus (LC), the amygdala (AMY) and the median raphe nucleus [6], all areas involved in supraspinal pain modulation [7]–[11]. In relation to receptor expression, GALR1 is greatly expressed in the LC, dorsal raphe nucleus (DRN), the paraventricular nucleus of the hypothalamus, DMH, AMY, thalamus and medulla oblongata [12]–[15]. However, in the AMY, GALR2/R3 are also significantly expressed [12]. Similarly, all types of GAL receptors are expressed in the prefrontal cortex and the hippocampus but to a lesser extent [12], [14], [15]. GALR2 is highly expressed in the hypothalamus, dentate gyrus, piriform cortex and mammillary nuclei [14], [15], while the expression of GALR3 has been reported mainly in the hypothalamus (preoptic, DMH, lateral and posterior hypothalamic, ventromedial and premammillary nuclei) [15], the bed nucleus of the stria terminalis, periaqueductal grey matter (PAG), lateral parabrachial nucleus and medial reticular formation [16]. Again, most brain areas mentioned above are involved in the codification and modulation of nociceptive inputs [7], [10].

The administration of exogenous GAL to the arcuate [17], tuberomammillary [18], nucleus accumbens [19], central nucleus of the AMY [20], [21] and PAG [22] decreases nociception in healthy rats, an effect that is mediated by GalR1 in rodents [23]. A similar effect is observed in some pathological conditions, such as acute inflammation or mononeuropathy [22], where the microinjection of supraspinal exogenous GAL also decreases nociception. Albeit the apparent antinociceptive role of supraspinal GAL in pain modulation, the intracerebroventricular administration of a GALR1 agonist in rats increased c-Fos expression in the DMH [24], an area that facilitates nociception by promoting behavioural hyperalgesia [9], [25]. As hyperalgesia is one of the hallmarks of chronic pain, activation of the DMH promotes behavioural hyperalgesia and GAL receptors are strongly expressed in the DMH, here we evaluated the contribution of GAL receptors in the DMH to the descending control of inflammatory hyperalgesia in monoarthritis as well as nociception in healthy controls.

## Methods

### 1. Animals, ethical issues and anaesthesia

The experiments were performed in adult male Wistar Han rats with 175–250 g (Charles Rivers, Barcelona, Spain). A total of 96 animals (SHAM, n = 48 and ARTH, n = 48) were used in the experiments herein, 40 animals (SHAM, n = 20 and ARTH, n = 20) were used in the behavioural assessment, 32 animals (SHAM, n = 16 and ARTH, n = 16) in the c-Fos protocol and 24 animals (SHAM, n = 12 and ARTH, n = 12) in the electrophysiological evaluation. Animals were randomly assigned two by two to boxes upon arrival; a blue line was painted in the tail of one rat and a red line in the tail of the other. Each box was numbered from 1 to 48, no indication concerning if the animals were assigned to the SHAM or ARTH group was displayed. The list discriminating the boxes corresponding to the SHAM or ARTH groups was kept by an independent party. Each animal was considered a single unit within its experimental group. Animals were housed two per cage, except for animals with chronic intracerebral cannulae implanted that were housed individually. Food and water were available *ad libitum* and animals were maintained in a climate-controlled room, under 22±2°C of temperature, 55±5% of humidity and under a 12 h light/dark cycle with lights on at 8:00am. The experimental protocol followed the European Community Council Directive 86/609/EEC and 2010/63/EU concerning the use of animals for scientific purposes and was approved by the Institutional Ethical Commission (Permit Number: 23248). All efforts were made to minimize animal suffering and to use only the number of animals necessary to produce reliable scientific data.

For cannula implantation the animals were anaesthetized i.p. with a mixture 1∶1.5 of ketamine (Imalgene, Merial, Oeiras, Portugal) and medetomidine (Dorbene, Esteve, Carnaxide, Portugal). After the surgical procedure, the anaesthesia was reversed using atipamezole (Antisedan, Pfizer, Oeiras, Portugal, i.p.) and the animals were monitored until fully awake (grooming and eating).

Anaesthesia was induced by administering pentobarbitone (50 mg/kg, i.p., Eutasil, CEVA, Algés, Portugal) and maintained by infusing pentobarbitone (15–20 mg/kg/h, i.p.). The level of anaesthesia was frequently assessed by determining behavioural responses to noxious pinching. Body temperature was maintained within physiological range with the help of a warming blanket (DC Temperature Controller, FHC, Bowdoin, ME, USA). At the end of the experiment, animals received a lethal dose of pentobarbitone.

### 2. Induction of arthritis

The induction of monoarthritis (ARTH) was performed four weeks before the actual experiments, as described in detail elsewhere [9], [26]. In order to maintain the researcher blind in relation to whether the animals from a specific box were assigned to the SHAM or ARTH groups, the animals were anaesthetized (section 2.1) by a third party in an adjacent room and then brought to the chirurgical table in groups of two for the injection of SAL or K/C in the right knee joint. Briefly, in anaesthetised animals a mixture of 3% kaolin and 3% carrageenan (K/C, Sigma-Aldrich, St. Louis, MO, USA) dissolved in saline was injected into the synovial cavity of the right knee joint at a volume of 0.1 mL. This model produces mechanical hyperalgesia, which begins a few hours after surgery and extends up to 8 weeks [27]. After the procedure, animals returned to the adjacent room, the anaesthesia was reversed and animals were monitored until fully recovered (eating and grooming). At the end of the induction session all boxes were returned to the animal house. In each animal, development of arthritis was verified again 1 h prior to each behavioural session. While confirming the arthritic status of the animals, through the flexion and extension of the right leg, the experimenter was handed the animals by a third party without any specific order and without prior knowledge of the box number. Only those rats that vocalized every time after five flexion–extension movements of the knee joint were considered to have arthritis, and they were included in the ARTH group. SHAM animals were injected with 0.1 mL saline in the synovial cavity of the right knee joint. SHAM animals did not vocalize to any of the five consecutive flexion–extension movements of the knee joint. After the test, the animals were returned to their home cages by a person other than the evaluator.

### 3. Behavioural assessment of nociception

All behavioural tests were performed during the day time, starting at 9:30am and ending at 1:30pm after which the animals were returned to the animal house.

#### 3.1 Mechanical hyperalgesia

The application of noxious pressure to the primary site of injury is a classical approach to measure mechanical hyperalgesia [28], both in humans and animals [29]. Here, the pressure application measurement (PAM; Ugo Basile, Comerio, Italy) method was used. It allows an accurate behavioural measurement of mechanical hypersensitivity in rodents with chronic inflammatory joint pain [30] by the application of a force range of 0–1500 g. To perform the test and with the animal securely held, the force transducer unit (fitted to the experimenter's thumb) is placed on one side of the animal's knee joint and the forefinger on the other and an increasingly force is applied across the joint until a behavioural response is observed (limb-withdrawal, freezing of whisker movement, wriggling or vocalization) with a cut-off of 5 s. The peak force applied immediately prior to the behavioural response is recorded as the response threshold (RT). RT was measured twice in the ipsilateral and contralateral limbs at 1 min intervals. The mean RTs were calculated per animal. At the end of the session animals were returned to their home cage.

#### 3.2 Thermal hyperalgesia (heat)

Heat hyperalgesia was evaluated using the Hargreaves test [31]. The rats were habituated to the experimental conditions by allowing them to spend 1–2 h daily in the experimental room for the three days preceding any behavioural tests [9]. For assessing heat hyperalgesia, a radiant heat source was placed under the hindpaws in awake animals and the time spent between the heat application and the withdrawal response (Plantar Test Instrument, Model 37370, Ugo Basile, Varese, Italy) was registered as the paw-withdrawal latency (PWL). In each session, the PWL was assessed prior to drug administration in the DMH and 20 min after. In each time point, the PWL was repeated twice at an interval of 1 min and the mean of these values was used in further calculations. Cut-off time was 15 s.

### 4. Procedures for intra-DMH microinjections

Before the placement of the guide cannulas the animals were anaesthetized (section 2.1) by a third party in an adjacent room and then brought to the chirurgical table one at the time. For intra-DMH drug administration, four weeks before the actual experiments (at the same time that arthritis was induced), animals were anaesthetised and placed in a stereotaxic frame, and one stainless steel guide cannula (26 gauge; PlasticsOne, Roanoke, VA, USA) was then implanted in the DMH according to the coordinates of the atlas by Paxinos and Watson [32]. The tip of the guide cannula was positioned 1 mm above the desired injection site in the DMH [AP, −3.24 mm from bregma; LM, 0.4 mm lateral from the midline (right side); DV, 7.5 mm below the surface of the skull]. The guide cannula was kept in place through the use of two dental screws and dental cement. A dummy cannula was inserted into the guide cannula to close the top. After the procedure, the anaesthesia was reversed and animals were monitored until being fully recovered (eating and grooming) in the adjacent room and returned to the animal house.

**Figure 1:**
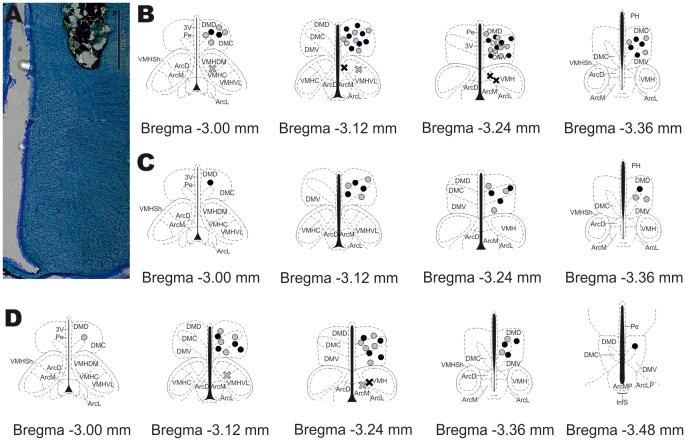
Anatomical confirmation of drug injection sites in the dorsomedial nucleus of the hypothalamus (DMH). (**A**) Photomicrograph of an example of the drug injection site in the right DMH of the rat brain (AP: −3.24 mm from bregma) superimposed with the appropriate plate of the Paxinos and Watson (2007) stereotaxic atlas. (**B**) Schematic representation of injection sites in the DMH during the behavioural study. The coordinates for the injection sites are as follow −3.00 mm, −3.12 mm, −3.24 mm and −3.36 mm from bregma. (**C**) Schematic representation of injection sites in the DMH during the protocol for the induction of c-Fos expression. The coordinates for the injection sites are as follow −3.00 mm, −3.12 mm, −3.24 mm and −3.36 mm from bregma. (**D**) Schematic representation of injection sites in the DMH during the electrophysiological study. The coordinates for the injection sites are as follow −3.00 mm, −3.12 mm, −3.24 mm, −3.36 mm and −3.48 mm from bregma. (Grey dots correspond to injection sites in the DMH of control (SHAM) animals and black dots show injection sites in the DMH of arthritic (ARTH) animals; grey and black crosses correspond to injection sites outside the DMH of SHAM and ARTH animals, respectively) ArcD- arcuate hypothalamic nucleus, dorsal; ArcL- arcuate hypothalamic nucleus, lateral; ArcM- arcuate hypothalamic nucleus, medial; DMC - dorsomedial hypothalamic nucleus, compact; DMD – dorsomedial hypothalamic nucleus, dorsal; DMV - dorsomedial hypothalamic nucleus, ventral; VMHDM - ventromedial hypothalamic nucleus, dorsomedial; VMHSh - ventromedial hypothalamic nucleus, shell; VMHVL - ventromedial hypothalamic nucleus, ventrolateral.

In order for the experimenter to remain blinded in relation to which animals were SHAMs or ARTHs, prior to the beginning of the behavioural session, the cards displaying the number of the box were substituted by cards displaying letters. Test drugs were administered in the DMH through a 33-gauge injection cannula (PlasticsOne) inserted into and protruding 1 mm beyond the tip of the guide cannula. The microinjection was made using a 10.0-µL-Hamilton syringe connected to the injection cannula by a polyethylene catheter (PE-10; Plastics One). The injection volume was 0.5 µL and therefore, the spread of the injected drugs within the brain was expected to be 1 mm [33]. The efficacy of injection was monitored by observing the movement of a small air bubble through the tubing. The injection lasted 20 s and the injection cannula was left in place for additional 30 s to minimize the return of drug solution back to the injection cannula. Brain injection sites were histologically verified from post-mortem sections and plotted on standardized sections from the stereotaxic atlas [32] (**Fig. 1**). After the completion of the tests and animals were returned to the animal house, the cards were switch again. The attribution of the letter cards was recorded in a lab book separate from the one used to register the results. The order of attribution of the letter cards was random and changed in each experimental session. The order of the administration of the drugs to each animal was defined at the beginning of the experiment to avoid potential confounding effects related to this parameter. The results of the tests were only associated with the respective animal after the end of the experiment.

### 5. Drugs

Solutions for drug administration in the DMH were prepared in sterilized saline 0.9% (Unither, Amiens, France; pH 7.2). All the experimental drugs used in this work were acquired from Tocris (Bristol, UK). Each injection had a volume of 0.5 µL and contained either GAL (1.0 nmol), a non-specific GAL receptor antagonist (M40, 1.0 nmol), a specific GALR1 agonist (M617, 1.0 nmol) or a specific GALR2 antagonist (M871, 1.0 nmol) [17], [20], [23]. Control injections were performed with SAL in order to avoid any confounding effect that might result from injecting the liquid itself.

### 6. Course of the pharmacological study

Four weeks following induction of arthritis and insertion of the guide cannula for DMH injections, the efficacy of DMH-induced phasic and tonic modulation of nociception was determined by assessing the effect of DMH injection of exogenous GAL, M40, M671 and M871 upon the PWL in awake SHAM and ARTH animals. SAL was used in control injections. The latency of the withdrawal response was assessed 20 min [18], [34] following the intra-DMH injections. The interval between behavioural assessments of different drug treatment conditions in the same animal was at least two days. The order of testing different drugs varied between the animals.

### 7. Recording of neuronal activity in nociceptive RVM cells

For the electrophysiological study, animals were removed from the animal house in a random order, one per day, already anaesthetized, by a person other than the experimenter. Anaesthesia (section 2.1) was administered at 9:30am, the electrophysiological recordings started between 10am and 10:30 am and lasted for 3 h. The order of the administration of the drugs varied between the animals. The electrophysiological recordings of the activity of RVM neurones followed a protocol described in Pinto-Ribeiro and colleagues [9]. In anaesthetised animals, a recording electrode was placed in the RVM (AP: 5.88 mm rostral to the interaural line, ML: −0.6 to 0.6 mm lateral from the midline, and DV: 10.0 mm below the surface of the skull) [32]. Single neurone activity was recorded extracellularly with tungsten electrodes (tip impedance 3–10 MΩ at 1 kHz), the signal was amplified and filtered and data sampling was performed through a CED Micro 1401 interface and Spike 2 software (Cambridge Electronic Design, Cambridge, UK).

Recording of RVM neurones was started after the animal was under light anaesthesia; i.e., the animals gave a brief withdrawal response to noxious pinch, but the pinch did not produce any longer lasting motor activity, nor did the animals have spontaneous limb movements. RVM neurones were classified based on their response to noxious heating of the tail with a tail-flick device (Ugo Basile). Heat stimulation of the tail was applied during 10 s. Functional classification of RVM neurones followed the scheme developed earlier by Fields and colleagues [35] and by Fields and Heinricher [36]. The neurones whose firing activity increased during heat stimulation of the tail were considered On-cells, those decreasing its activity were classified as Off-cells and finally, cells displaying only a negligible (<10%) or no alteration in discharge rates during noxious stimulation were considered Neutral-cells and were not analysed in this study. However, a significant difference with the classification scheme of Fields [35] is that in the present study the noxious stimulus-induced withdrawal reflex was not taken into account in the classification. Therefore, as in previous studies, RVM cells are here called On-like and Off-like cells [9], [37], [38] rather than On- or Off-cells.

The characterization of the response properties of RVM cells consisted of the following assessments performed successively: (i) spontaneous activity; (ii) response to heating of the tail; (iii) recovery to the spontaneous activity level.

It should be noted that when analysing responses of RVM neurones to peripheral stimulation, the baseline discharge frequency (recorded just before the stimulation) was subtracted from the discharge frequency assessed during the stimulation using the following formula:
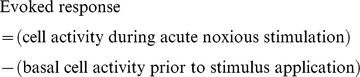



Thus, positive values represent an increase and negative ones represent a decrease in cell activity evoked by peripheral stimulation.

During the recordings, animals also had a guide cannula implanted for drug administration into the DMH. After determining the baseline spontaneous activity of RVM cells and their baseline noxious-evoked responses to peripheral stimulation, either exogenous GAL or a non-specific GALRs antagonist (M40) were microinjected in the DMH, in order to assess its phasic or tonic effect, respectively, upon the discharge rate of RVM neurones. All results from drug administrations were plotted for the variation in activity comparing baseline (before drug administration) and values obtained 20 min after the injection into the DMH. The results of the electrophysiological analysis were only associated with the respective animal after all recordings were performed.

### 8. Course of the electrophysiological study

Electrophysiological recordings of RVM neurones (SHAM: On-like cells, n = 58 and Off-like cells, n = 40; ARTH: On-like cells, n = 46 and Off-like cells, n = 47) were performed under pentobarbitone anaesthesia four weeks after the administration of K/C (ARTH) or SAL (SHAM) in the right knee of animals. In RVM recordings, the response properties of nociceptive neurones were assessed by determining their spontaneous activity and the response to noxious heating of the tail. Search for the next neurone to be studied started about 30 min after testing of the previous one was completed. At the end of the recording session, electrolytic lesions were made in the recording sites, the animals were given a lethal dose of pentobarbitone and the brains were removed for histological verification of the recording and injection sites.

### 9. c-Fos study

**Figure 2:**
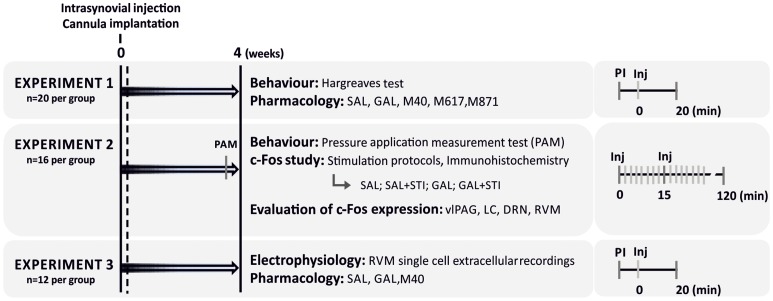
Schematic representation of the experimental design. In all experiments, animals were divided in two groups, control (SHAM) when injected with saline and arthritic (ARTH) when injected with a mixture of kaolin and carrageenan in the synovial capsule of the right knee joint. Three days after the intrasynovial injection, arthritis was confirmed by performing five consecutive movements of flexion/extension of the knee (dashed line). Animals in the ARTH group developed a clear swelling of the treated knee joint and all gave a vocalization response during a minor extension and flexion of the affected limb by the experimenter. SHAM animals displayed no obvious swelling of the knee joint and did not vocalize when the limb was flexed. Four weeks after the induction of monoarthritis animals were tested in three independent experiments. In experiment 1, the Hargreaves test was used to study the effect of exogenous galanin (GAL), a non-specific GAL receptor antagonist (M40), a specific GAL receptor-1 agonist (M617) and a specific GAL receptor-2 antagonist (M871) in the dorsomedial nucleus of the hypothalamus (DMH) upon paw-withdrawal latency (PWL) (n = 20 per experimental group). In each animal, the development of arthritis was confirmed again 1 h prior to each behavioural session by performing five consecutive movements of flexion/extension of the knee. During the experimental sessions, PWL was assessed before and 20 min after the administration of the drugs to the DMH. In experiment 2, two days prior the c-Fos study, the pressure application measurement (PAM) test was performed to confirm the arthritic state of the animals. c-Fos expression was evaluated in SHAM and ARTH animals after exogenous GAL or saline (SAL) administration in the DMH, peripheral noxious mechanical stimulation and the simultaneous application of noxious mechanical stimulation after the microinjection of exogenous GAL in the DMH (n = 16 per experimental group). Peripheral stimulation was applied each 2 minutes during 2 hours and two drug injections were made in the DMH, one at the beginning and another 15 minutes after the beginning of peripheral stimulation. Neurones expressing c-Fos were quantified bilaterally in the ventrolateral periaqueductal grey matter (vlPAG), locus coeruleus (LC), dorsal raphe nucleus (DRN) and rostral ventromedial medulla (RVM). In experiment 3, RVM neurones were recorded before and after the administration of exogenous GAL and M40 in the DMH. The assessment of neuronal activity includes a preliminary evaluation of spontaneous and noxious-evoked activity followed by the recording of these parameters 20 min after drug administration to the DMH (n = 12 animals per experimental group). PI – Pre-injection; Inj – Injection; SAL - saline microinjection in the dorsomedial nucleus of the hypothalamus; GAL - galanin microinjection in the dorsomedial nucleus of the hypothalamus; SAL+STI – saline microinjection in the dorsomedial nucleus of the hypothalamus and extension of right limb; GAL+STI – galanin microinjection in the dorsomedial nucleus of the hypothalamus and extension of right limb.

For the c-Fos induction protocol, animals were removed from the animal house in a random order, one per day, already anaesthetized, by a person other than the experimenter. Anaesthesia (section 2.1) was administered at 9:30 am; the protocol started between 10am and 10:30am and lasted for 2 h. To evaluate changes in brain activation after exogenous GAL administration in the DMH and/or peripheral noxious stimulation in SHAM and ARTH animals, c-Fos immunoreaction was performed following the protocol described elsewhere [39]. Animals were held in a stereotaxic frame. For drug administration, a guide cannula was placed in the DMH according to the coordinates of the atlas by Paxinos and Watson [32] and one of the following protocols was performed: (i) SAL microinjection in the DMH of SHAM animals; (ii) exogenous GAL microinjection in the DMH of SHAM animals; (iii) SAL microinjection in the DMH and extension of right limb of SHAM animals; (iv) exogenous GAL microinjection in the DMH and extension of right limb of SHAM animals; (v) SAL microinjection in the DMH of ARTH animals; (vi) exogenous GAL microinjection in the DMH of SHAM animals; (vii) extension of right limb of ARTH animals; (viii) exogenous GAL microinjection in the DMH and extension of right limb of ARTH animals. Two exogenous GAL (or SAL) doses were injected in the DMH with a 15 min interval (**Fig. 2**). Extension of the paw was performed 5 times every 2 minutes for 2 h. Two hours after the first injection and first knee extension (beginning of the protocol), the animals were transcardially perfused with 4% paraformaldehyde in 0.1 M phosphate buffer saline (PBS, pH = 7.4), brains were removed and then post-fixed overnight in the same fixative and kept in a solution of 8% sucrose in PBS. One in three coronal vibratome (Leica, Carnaxide, Portugal) sections (50 µm thick) were treated with a solution of 3.3% H2O2 in PBS (30 min) to inhibit endogenous peroxidase activity, and then sequentially washed thrice (10 min) in PBS and PBS-Triton (PBS-T; 0.3% triton X-100; Sigma-Aldrich, Sintra, Portugal). Sections were then incubated in a blocking solution of 2.5% fetal bovine serum (FBS; Biochrom, Cambridge, United Kingdom) in PBS for 2 h, followed by the incubation overnight at 4°C in rabbit anti-Fos antibody (1∶2000 in PBST and 2% FBS; Calbiochem, Merck, Algés, Portugal). The following day, after three washes (10 min) in PBST, sections were incubated in biotinylated polyclonal swine anti-rabbit antibody (1∶200 in PBST; Dako, Denmark) for 1 h and again washed thrice (10 min) in PBS-T. Sections were then incubated in avidin–biotin complex (ABC; 1∶200 in PBST; Vectastain, Vector Laboratories, Peterborough, USA) for 1 h followed by a series of washing steps with PBST (twice, 10 min), PBS (twice, 10 min) and Tris-HCl (0.05 M, pH 7.6) (twice, 10 min). Finally, sections were stained with diaminobenzidine (0.0125% in a solution of Tris-HCl with 0.02% H2O2; Sigma Aldrich) and washed twice (10 min) with Tris-HCl and PBS. After staining, the sections were mounted on SuperFrost slides (Braunschweig, Germany). c-Fos levels were determined by counting the number of Fos-immunoreactive neurones occurring bilaterally in the brainstem with the aid of a Stereo Investigator 10 Software (Microbrigthfield Bioscience, Madgedurg, Germany) using a video camera (Microbrigthfield Bioscience) attached to a microscope (BX51, Olympus Iberia, Lisboa, Portugal).

### 10. Statistics

For the effect of drugs upon PWL, the minimum number of animals needed was determine à priori using the G power software (version 3.1.9.2, University of Kiel, Germany) considering a ANOVA-2-way test, α err probability of 0.05, power of 0.95 and an effect size of 0.80 was n = 23. For the effect of drugs upon RVM neuronal activity, the minimum number of animals needed was determine à priori using the G power software considering a ANOVA-2-way test, α err probability of 0.05, power of 0.95 and an effect size of 0.80 was n = 28. For the effect of drugs upon c-Fos expression, the minimum number of animals needed was determine à priori using the G power software considering a ANOVA-2-way test, α err probability of 0.05, power of 0.95 and an effect size of 0.80 was n = 32. The results of the RT analysis correspond to the mean ± SD of raw data; no method of data normalization was used. To assess the effect of the drugs upon PWL for each behavioural session, the value of the basal withdrawal latency (withdrawal latency prior to drug administration) was subtracted from the value of the withdrawal latency at the peak effect of the drug, a negative value indicated the withdrawal latency decreased while a positive value corresponded to an increase in withdrawal latency after drug administration to the DMH. To perform this evaluation, raw data was used. To assess the effect of the drugs upon spontaneous neuronal activity, the value of the activity of RVM On- and Off-like cells without noxious peripheral stimulation prior to the administration of drugs in the DMH was subtracted from the activity of these cells without noxious peripheral stimulation at the peak effect of the drug. Similarly, to assess the effect of the drugs upon the noxious-evoked neuronal activity the value of the activity of RVM On- and Off-like cells during noxious peripheral stimulation prior to the administration of drugs in the DMH was subtracted from the activity of these cells during noxious peripheral stimulation at the peak effect of the drug. Only raw data was used in this analysis. To compare the level of c-fos in each area, the total number of cells stained was registered per area studied and only raw data was used in this analysis. The GraphPad Prism 6 software (GraphPad Software Inc, La Jolla, CA, USA) was used to perform the statistical analysis. The comparison of differences between RT in the PAM test and between the baseline of RVM neuronal spontaneous and heat-evoked activities of SHAM and ARTH animals were performed using a student's t-test for unpaired data. To compare differences in RT between the ipsilateral and the contralateral side in SHAM and ARTH animals a student's t-test for paired data was used. All other comparisons between groups were performed using a two-way ANOVA followed by a Bonferroni correction for multiple comparisons post-hoc test. Statistical significance was accepted for *P*<0.05.

## Results

### 1. Monoarthritic animals developed ipsilateral mechanical allodynia

Three days after the intrasynovial injection, all animals in the ARTH group developed a clear swelling of the treated knee joint and all gave a vocalization response during a minor extension and flexion of the affected limb by the experimenter. SHAM animals displayed no obvious swelling of the knee joint and did not vocalize when the limb was flexed.

**Figure 3:**
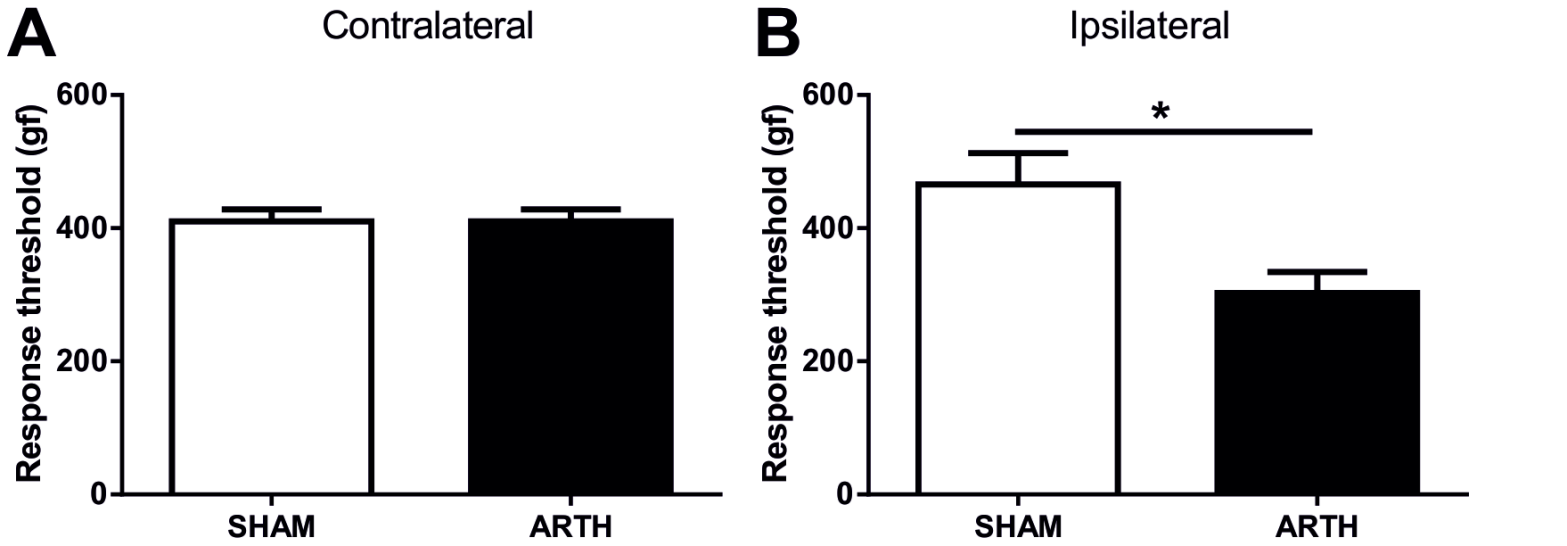
Response threshold. Four weeks after the induction of monoarthritis in the right hind limb, no differences were observed in the response threshold (RT) of the contralateral hindpaws between control (SHAM) and arthritic (ARTH) animals (**A**) in the pressure application measurement. In the ipsilateral side however ARTH animals displayed a decrease in RT during the pressure application measurement when compared to SHAM animals (**B**). Mean response threshold is presented as mean + SEM. (*P<0.05, t-test for unpaired data). gf – gram force.

Mechanical hyperalgesia in the knee joint was assessed by determining RT to mechanical pressure over the knee joint. No differences were found between the RT of the ipsilateral and contralateral hindpaws in SHAM animals (*t*_7_ = 1.535, *P* = 0.169) while in ARTH animals the ipsilateral RT was significantly lower than the contralateral (*t*_7_ = 3.377, *P* = 0.0118). No differences were found between the contralateral RT of SHAM and ARTH animals (*t*_14_ = 0.000, *P*>0.999). Four weeks after induction of monoarthritis, RT was significantly different between SHAM and ARTH animals (*t*_14_ = 2.883, *P* = 0.012). This result indicates that K/C induced a significant RT decrease, i.e., mechanical hyperalgesia (**Fig. 3**).

### 2. Exogenous GAL in the DMH decreases paw-withdrawal latency, an effect reversed by the administration of a GAL receptors antagonist

**Figure 4:**
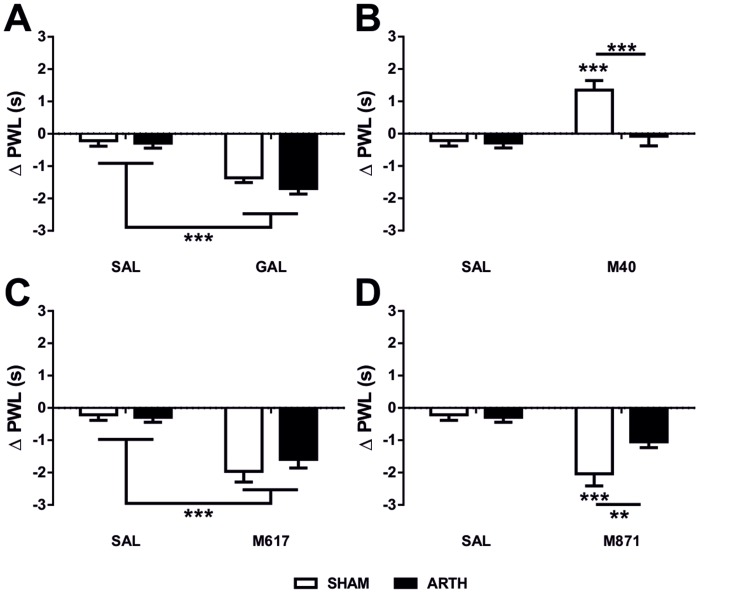
Paw-withdrawal latency after drug administration in the dorsomedial nucleus of the hypothalamus (DMH). In this experiment the analysis of the paw-withdrawal latencies in control (SHAM) and arthritic (ARTH) animals was performed 20 minutes after the intracerebral microinjection of either exogenous galanin (GAL) (**A**), a non-specific antagonist of GAL receptors (M40; **B**), a specific agonist of GAL receptor-1 (M617; **C**) or a specific antagonist of GAL receptor-2 (M871; **D**). Note that the pronociceptive action of exogenous GAL administration in the DMH in SHAM and ARTH animals (**A**) is only mimicked by the microinjection of M617 (**C**). Mean response latency is presented as mean + SEM. (*P<0.05, **P<0.01, ***P<0.001, t-test with a Bonferroni correction for multiple comparisons).

To investigate a possible role of supraspinal GAL in phasic and tonic pain facilitation in SHAM and ARTH animals, paw withdrawal latencies (PWL) were assessed after exogenous GAL or M40 microinjection, respectively, in the DMH. The PWL of SHAM and ARTH animals 20 min after exogenous GAL microinjection in the DMH was significantly decreased when compared with SAL injection (main effect of the drug: *F*_1,76_ = 61.880, *P*<0.001). The exogenous GAL-induced decrease in PWL was of the same magnitude in the SHAM and ARTH groups (main effect of the group: *F*_1,76_ = 2.704, *P* = 0.104). *Post hoc* tests confirmed that the PWL of SHAM and ARTH animals treated with exogenous GAL was significantly lower than the PWL of SHAM and ARTH animals treated with SAL (**Fig. 4A**).

Non-specific inhibition of GAL receptors induced by administration of M40 in the DMH significantly altered the PWL when compared with SAL injection (main effect of the drug: *F*_1,76_ = 13.830, *P*<0.001). The effect of M40 was significantly different between SHAM and ARTH animals (main effect of the group: *F*_1,76_ = 10.070, *P* = 0.002). The M40-induced effect on PWL varied with the experimental group (interaction between group and drug: *F*_1,76_ = 8.048, *P* = 0.006). *Post hoc* tests indicated that M40 significantly increased PWL in SHAM animals, but did not alter PWL in ARTH animals (**Fig. 4B**).

### 3. Nociceptive facilitation after exogenous GAL in the DMH is mediated by GAL receptors type-1

To determine which GAL receptor is involved in pain facilitation induced by exogenous GAL in the DMH, PWL was assessed after the administration of M617 (a specific agonist of GAL receptor type-1 - GalR1) and M871 (a specific antagonist of GAL receptor type-2 - GalR2) into the DMH. Twenty minutes after microinjecting M617 in the DMH, PWL was significantly decreased when compared with SAL injection (main effect of the drug: *F*_1,76_ = 39.530, *P*<0.001). The effect of M617 was not different between SHAM and ARTH groups (main effect of the group: *F*_1,76_ = 0.357, *P* = 0.552). *Post hoc* tests confirmed that PWL significantly decreased after M617 administration in the DMH both in SHAM and ARTH animals when compared to PWL after SAL administration (**Fig. 4C**).

Administration of M871 in the DMH had a significant effect on PWL (main effect of the drug: *F*_1,76_ = 29.820, *P*<0.001), and the effect of M871 varied with the experimental group (interaction group x drug: *F*_1,76_ = 5.089, *P* = 0.027). *Post hoc* tests showed that M871 significantly decreased the PWL in SHAM animals but did not alter significantly the PWL of ARTH animals (**Fig. 4D**).

### 4. Expression of c-Fos in brainstem areas involved in pain control is altered by exogenous GAL in the DMH

Descending pain modulatory drive from the forebrain to the spinal cord may be relayed by multiple areas in the brainstem. To determine which brainstem areas mediate exogenous GAL-driven descending pain modulatory effects originating in the DMH, c-Fos expression was investigated in caudal brain areas that not only expressed GAL and/or its receptors but that are also involved in the descending modulation of nociception. Hence, we compared changes in c-Fos expression in the ventrolateral periaqueductal grey matter (VLPAG), the LC, the dorsal raphe nucleus (DRN) and the rostral ventromedial medulla (RVM) between SHAM and ARTH animals after (i) SAL microinjection in the DMH of SHAM animals; (ii) GAL microinjection in the DMH of SHAM animals; (iii) SAL microinjection in the DMH and extension of right limb of SHAM animals; (iv) GAL microinjection in the DMH and extension of right limb of SHAM animals; (v) SAL microinjection in the DMH of ARTH animals; (vi) GAL microinjection in the DMH of SHAM animals; (vii) extension of right limb of ARTH animals; (viii) GAL microinjection in the DMH and extension of right limb of ARTH animals.

**Figure 5:**
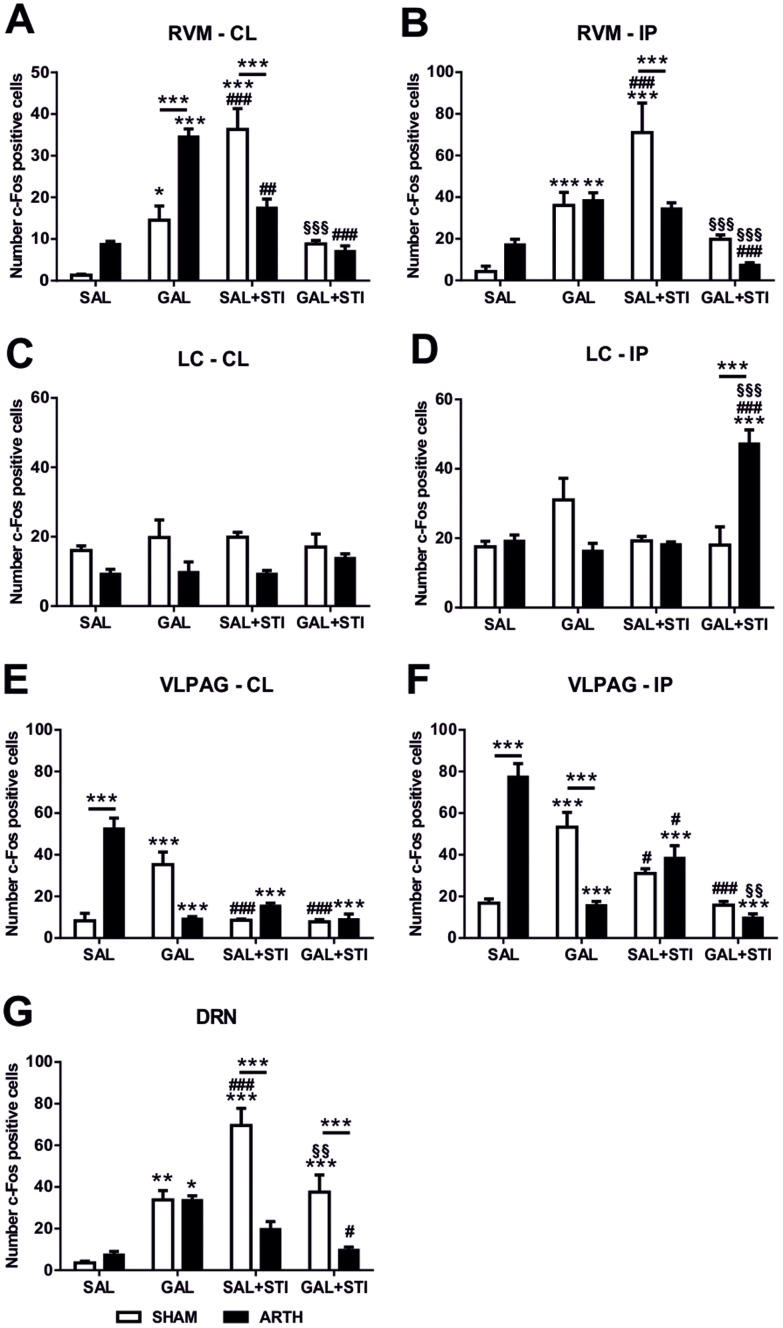
Brainstem c-Fos expression. Number of c-Fos positive cells in the contralateral (**A,C,E**) and ipsilateral (**B,D,F**) sides of the rostral ventromedial medulla (RVM; **A,B**), locus coeruleus (LC; **C,D**), ventrolateral periaqueductal matter (vlPAG; **E,F**) and dorsal raphe nucleus (DRN; **G**) after the administration of saline or exogenous galanin in the dorsomedial nucleus of the hypothalamus (DMH) with and without peripheral noxious stimulation. Data is presented as mean + SEM. SAL – Saline; GAL – Galanin; SAL+STI – SAL and peripheral noxious stimulation (limb flexion-extension); GAL+STI – GAL and peripheral noxious stimulation (limb flexion-extension). * indicates significant differences in c-Fos expression when compared to SAL injection in the DMH; * over line indicates differences in c-Fos expression between experimental groups; # indicates significant differences in c-Fos expression when compared to GAL injection in the DMH; § indicates significant differences in c-Fos expression when compared to the flexion-extension protocol (*, ^#^ *P*<0.05; **, ^##^, ^§§^ *P*<0.01; ***, ^###^, ^§§§^ *P*<0.001).

Expression of c-Fos following injection of SAL in the DMH was considered to represent basal activation. The number of c-Fos positive neurones in the contralateral RVM varied with the stimulation protocol used (main effect of the protocol: *F*_3,24_ = 22.570, *P*<0.001) with different effects on SHAM and ARTH animals (interaction group x protocol: *F*_3,24_ = 42.280, *P*<0.001). *Post-hoc* testing showed that exogenous GAL in the DMH significantly increased c-Fos expression when compared to SAL-injected animals in both experimental groups, although a higher expression was observed in ARTH animals (**Fig. 5A**). The flexion-extension protocol (SAL+STI) increased c-Fos expression in SHAM animals when compared to SAL and GAL administration while it decreased its expression in ARTH animals when compared to GAL-Injected ARTH. The simultaneous infusion of GAL in the DMH and flexion-extension of the injected limb decreased c-Fos expression in SHAM when compared with its expression after the flexion-extension protocol and in ARTH when compared with GAL-injected ARTH (**Fig. 5A**). Similarly, in the ipsilateral RVM, the number of cells activated was significantly different depending on the protocols (main effect of the protocol: *F*_3,24_ = 70.240, *P*<0.001), an effect that varied with the experimental group (interaction group x protocol: *F*_3,24_ = 20.240, *P*<0.001). *Post-hoc* testing showed that GAL in the DMH increased the number of c-Fos expressing cells in both experimental groups when compared to SAL-injected animals, while its expression was different between SHAM and ARTH animals after repeated flexion-extension of the injected limb, with increased c-Fos expression in SHAM animals alone when compared with SAL and GAL-injected SHAM (**Fig. 5B**). The simultaneous injection of GAL in the DMH and flexion-extension of the injected limb significantly decreased the number of c-Fos positive cells in SHAM when compared to the flexion-extension protocol and in ARTH animals when compared with GAL administration and the flexion-extension protocols (**Fig. 5B**).

The number of c-Fos expressing neurones in the contralateral LC did not vary with the stimulation protocols (main effect of the protocol: *F*_3,24_ = 0.413, *P* = 0.745) although it was significantly different between experimental groups (main effect of the group: *F*_3,24_ = 16.410, *P*<0.001). *Post-hoc* testing did not show a specific alteration between each stimulation protocol (**Fig. 5C**). In the ipsilateral LC, the number of c-Fos positive cells varied with the stimulation protocol (main effect of the protocol: *F*_3,24_ = 7.462, *P* = 0.001), an effect that depended on the experimental group (interaction group x protocol: *F*_3,24_ = 14.310, *P*<0.001). *Post-hoc* testing showed an increase in the number of c-Fos expressing neurones after the simultaneous infusion of GAL in the DMH and flexion-extension of the injected limb in ARTH animals when compared with the same protocol in SHAM and with the SAL/GAL/flexion-extension protocols in ARTH (**Fig. 5D**).

In the contralateral vlPAG, the number of c-Fos expressing cells varied with the stimulation protocol (main effect of the protocol: *F*_3,24_ = 19.200, *P*<0.001), an effect that depended on the experimental group (interaction group x protocol: *F*_3,24_ = 40.030, *P*<0.001). *Post-hoc* testing showed a significant increase in the number of c-Fos positive cells in ARTH animals when compared to SHAM and after exogenous GAL in the DMH of SHAM animals when compared to SAL injected SHAM (**Fig. 5E**). In addition, in ARTH animals the number of c-fos expressing neurones was significantly lower in all protocols when compared to SAL injected ARTH animals (**Fig. 5E**). Similarly, the number of cells activated in the ipsilateral vlPAG was different after the stimulation protocols (main effect of the protocol: *F*_3,24_ = 22.920, *P*<0.001) and depended on the experimental group (interaction group x protocol: *F*_3,24_ = 47.100, *P*<0.001). *Post-hoc* testing (**Fig. 5F**) showed increased c-Fos expression in SAL-injected ARTH animals when compared with SAL-injected SHAM. GAL in the DMH significantly increased c-Fos expression in SHAM animals while it significantly decreased its expression in ARTH animals. c-Fos expression after the flexion-extension of the injected limb was not significantly different when compared to SAL-injected SHAM although it was significantly decreased when compared to GAL-injected SHAM (**Fig. 5F**). This protocol also significantly decreased c-Fos expression in ARTH animals when compared to SAL-injected ARTH although its expression was significantly higher when compared to GAL-injected ARTH. The simultaneous infusion of GAL in the DMH and flexion-extension of the injected limb did not significantly alter c-Fos expression when compared to SAL-injected SHAM but was significantly decreased when compared to its expression after GAL injection in the DMH. In ARTH animals, c-Fos expression was significantly decreased after the simultaneous infusion of GAL in the DMH and flexion-extension of the injected limb when compared to SAL-injected ARTH and the flexion-extension protocol (**Fig. 5F**).

Finally, In the DRN, the number of c-Fos positive cells varied with the stimulation protocol (main effect of the protocol: *F*_3,24_ = 24.690, *P*<0.001) and this effect was dependent of the experimental group (interaction group x protocol: *F*_3,24_ = 14.140, *P*<0.001). *Post-hoc* testing showed an increased DRN activation after GAL in the DMH in both experimental groups when compared to SAL-injected animals (**Fig. 5G**). The flexion-extension of the injured limb significantly increased c-Fos expression in SHAM animals when compared to ARTH animals and when compared with SAL- and GAL-injected SHAM. The simultaneous infusion of GAL in the DMH and flexion-extension of the injected limb increased c-Fos expression in SHAM animals when compared to ARTH and to its expression after SAL, but decreased when compared to the flexion-extension protocol. Additionally, it decreased c-Fos expression in ARTH animals when compared to GAL-injected ARTH (**Fig. 5G**).

### 5. The activity of pain modulatory On- or Off-like cells in the RVM is not altered by exogenous GAL in the DMH

To evaluate the effect of exogenous GAL administration in the DMH upon the activity of RVM neurones, the spontaneous and heat-evoked activities of presumably pronociceptive RVM On-like cells and antinociceptive RVM Off-like cells were recorded in SHAM and ARTH animals before and after the administration of exogenous GAL, M40 or SAL.

Before drug administration, the spontaneous activity of RVM On-like cells was significantly decreased in ARTH animals when compared to SHAM animals (**Table 1**). The magnitude of the response evoked by noxious heating of the tail in RVM On-like cells was not different between SHAM and ARTH animals. In RVM Off-like cells, the spontaneous activity before drug treatments was significantly decreased in ARTH animals when compared to SHAM animals. Similarly, the magnitude of the heat-evoked response in RVM Off-like cells of ARTH animals was significantly lower when compared to that in SHAM animals (**Table 1**).

**Table 1:** Spontaneous and heat-evoked baseline activities of rostral ventromedial medullary (RVM) On- and Off-like cells.

	Cell type	SHAM	ARTH	*t*	*P*
**Spontaneous**	**On-like**	**1.86±0.39 Hz**	**0.77±0.19 Hz**	***t*** **_102_ = 2.689**	***P*** ** = 0.008** **
	**Off-like**	**4.77±0.46 Hz**	**3.37±0.26 Hz**	***t*** **_85_ = 2.833**	***P*** ** = 0.006** **
**Evoked**	**On-like**	**2.51±0.31 Hz**	**2.82±0.37 Hz**	***t*** **_102_ = 0.601**	***P*** ** = 0.549**
	**Off-like**	**-2.45±0.25 Hz**	**-1.72±0.26 Hz**	***t*** **_85_ = 2.782**	***P*** ** = 0.007** **

Data presented as mean ± SEM.

**P<0.01.

**Figure 6:**
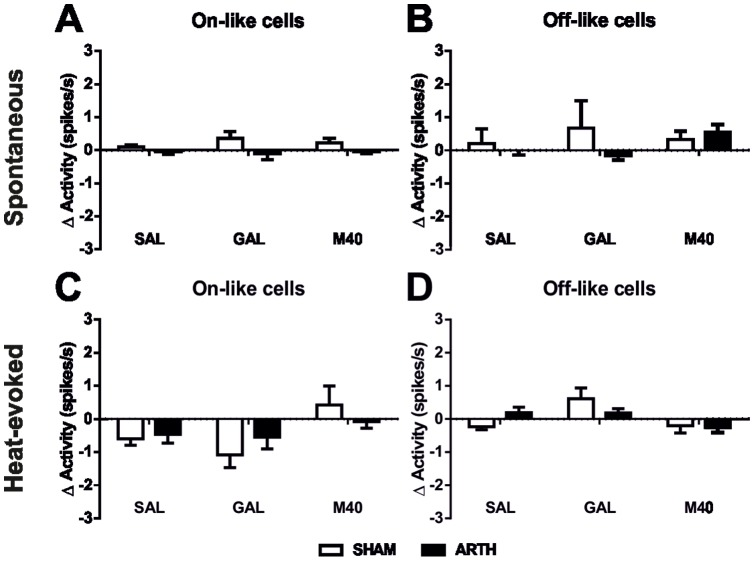
Spontaneous and noxious-evoked rostral ventromedial medulla (RVM) cell activity after the administration of exogenous galanin (GAL) or a non-selective GAL receptor antagonist (M40) in the dorsomedial nucleus of the hypothalamus (DMH). Overall, no changes were observed in the spontaneous (**A, B**) and noxious-evoked (**C, D**) activity of RVM pronociceptive On-like (**A, C**) and antinociceptive Off-like cells (B,D) before and 20 min after the intracerebral microinjection of exogenous GAL (**A–D**) and M40 (**A–D**). Data is presented as mean + SEM.

Microinjection of drugs into the DMH did not alter the spontaneous activity of RVM On-like cells (main effect of the drug: *F*_2,98_ = 0.262, *P* = 0.770). Overall, after drug injection, On-like cell spontaneous activity was different between ARTH and SHAM animals (main effect of the group: *F*_1,98_ = 6.510, *P* = 0.012) (**Fig. 6A**), although *post-hoc* tests failed to show a significant difference between experimental groups at a specific time point. The administration of drugs to the DMH did not alter the spontaneous activity of RVM Off-like cells (main effect of the drug: *F*_2,81_ = 0.616, *P* = 0.543) and the spontaneous activity was not different between experimental groups (main effect of the group: *F*_1,81_ = 1.200, *P* = 0.277) (**Fig. 6B**) 20 min after drug administration.

Microinjection of drugs into the DMH altered the heat-evoked activity of RVM On-like cells (main effect of the drug: *F*_2,98_ = 5.010, *P* = 0.009) but this effect did not vary with the experimental group (interaction group x drug: *F*_2,98_ = 1.318, *P* = 0.272). *Post hoc* testing failed to find significant drug treatment-induced effects on the heat-evoked response of On-like cells (**Fig. 6C**). Similarly, drug administration in the DMH changed the heat-evoked activity of RVM Off-like cells (main effect of the drug: *F*_2,81_ = 4.967, *P = *0.009) but these differences did not vary with the experimental group (interaction group x drug: *F*_2,81_ = 2.230, *P* = 0.114), 20 min after administration. Again, *post hoc* testing failed to find significant drug treatment-induced effects on the heat-evoked response of Off-like cells, except for the increase of response after exogenous GAL treatment in the SHAM group (**Fig. 6D**).

## Discussion

This study demonstrates, for the first time, a pronociceptive role for supraspinal GAL, as the administration of this neuropeptide to the DMH significantly increased spinal nociception (as indicated by the decrease in PWL) in awake healthy and arthritic animals. Moreover, the microinjection of GAL receptor agonist/antagonist in the DMH showed that the exogenous GAL's pronociceptive effect was mediated by GALR1 but not GALR2. The analysis of c-Fos expression revealed the serotonergic RVM and DRN, particularly in SHAM animals, as caudal areas potentially involved in signalling this descending pronociceptive effect. The exogenous GAL-induced increase of c-Fos expression in the RVM may not be explained by action on RVM On-like or Off-like pain modulatory cells, as the discharge rates of these two non-serotonergic cell types remained unaltered during pharmacological manipulations in the present study.

### 1. Novel pronociceptive effect of supraspinal GAL

Administration of exogenous GAL into the DMH induced behavioural hyperalgesia (decreased PWL) in healthy and ARTH animals. This is a novel effect for GAL as previous studies had only reported an antinociceptive role of this neuropeptide after its administration in brain areas involved in pain modulation, such as the hypothalamic arcuate nucleus [40], central AMY [20] and the PAG [22]. Thus, and similarly to what is observed at the spinal cord level [41], [42], GAL appears to have a bidirectional role in supraspinal descending pain modulation depending on the area where GALRs are activated. The demonstration of a tonic pronociceptive effect of GAL, by treatment of the DMH with a non-specific GALR antagonist, supports the proposal that the pronociceptive effect of GAL was mediated by GALRs.

Administration of exogenous GAL in the DMH facilitated nociception in both ARTH and SHAM animals. This finding contrasts with the results of previous studies indicating that exogenous GAL is antinociceptive when administered in the hypothalamic arcuate nucleus of animals with inflammation [17], or in the PAG of animals with mononeuropathy [22]. Importantly, the present results show that the descending GAL-driven pathway originating in the DMH, unlike the glutamate-driven pathway [9], remains functional in animals with experimental monoarthritis. Administration of a non-specific GALR antagonist alone into the DMH of ARTH animals had no effect on nociception while it produced antinociception in SHAM controls. This finding suggests that the GAL-driven pathway descending from the DMH is not tonically active in ARTH as in SHAM animals, but its activation in ARTH animals depends on the activation of upstream pathways inducing the release of GAL in the DMH.

### 2. GAL-driven nociceptive facilitation is mediated by GALR1

Further analysis on the contributions of GALR1 and GALR2 to the pain modulatory role of GAL in the DMH demonstrated that the facilitatory effect of GAL is mediated by GALR1, a receptor that couples to the Gi/Go pathway to decrease adenylyl cyclase activity [43]. Once more, this result contrasts with the available literature, where the activation of this receptor at spinal and supraspinal levels is reported to elicit an antinociceptive effect [44]–[46]. In fact, in the spinal cord it was GALR2 that has been reported to have a pronociceptive effect [41]; however, the results on administration of a GALR2 antagonist in the present study indicated that endogenous GAL acting on GALR2 had a tonic antinociceptive action in SHAM animals, whereas blocking GALR2 did not alter nociception in ARTH animals. Another possibility would be that the differential distribution of GALR1 and GALR2 receptors in the DMH could contribute to enhance GALR1-dependent effects, however as demonstrated by Mitchell and collaborators [47], not only does mRNAs analysis confirm an overlapping of GAL-R1 and GAL-R2 in the DMH but both receptors are also highly expressed in this nucleus. On the other hand, the expression of both receptors in the DMH does not account *per se* for the GAL/DMH pronociceptive effect since these receptors are also highly expressed and overlapping in the arcuate nuclei, an area where the intracerebral administration of exogenous GAL promotes antinociception [17]. Overall, it is probable that the facilitation of nociceptive behaviour by GAL in the DMH of ARTH animals results (i) from disinhibition of pronociceptive pathways driven by GALR1 and/or (ii) from a decrease in the activity of antinociceptive GALR2-driven circuits.

It is also possible that behavioural hyperalgesia in ARTH animals is reinforced by their emotional-like status. A recent study from our group [48] showed that animals with experimental monoarthritis displayed depressive-like behaviour. Interestingly, Blackshear *et al.* [24] showed that the intracerebroventricular injection of GAL and M617 increased c-fos expression in the DMH and the AMY, a nuclei involved the modulation of the emotional component of pain. Another work [49] showed that acute activation of GALR1 promoted the expression of ‘prodepressive-like’ behaviours, while GALR2 mediated the ‘antidepressant-like’ effects of GAL. Hence, taking into account that depressive states heighten pain perception in humans [50] and rodents [51], the pronociceptive GALR1 and the antinociceptive GALR2 effects observed in this study may be related to comorbid mood alterations known to be associated with chronic pain [52], [53].

### 3. Activation of serotonergic nuclei is influenced both by exogenous GAL in the DMH and noxious peripheral stimulation

The analysis of c-Fos expression was restricted to the VLPAG, DR, LC and RVM since these areas have been previously demonstrated to be strongly modulated by the DMH [54]–[56], while simultaneously implicated in nociceptive processing [57]–[59]. The limb extension-induced increase in c-Fos expression in the VLPAG and RVM of SHAM animals suggests that repetitive extension of a non-arthritic knee joint for a period of two hours can be considered a noxious stimulus [60]. In addition, the increased c-for expression in the VLPAG and RVM also suggests that repetitive knee joint extension activated the feedback loop of nociception involving the PAG-RVM-spinal dorsal horn circuitry, which may either inhibit or facilitate nociception [11], [61], [62].

Administration of exogenous GAL into the DMH increased the expression of c-Fos ipsilaterally in the VLPAG and bilaterally in the RVM, which suggests that DMH neurones expressing GALR are able to activate descending nociceptive controls. However, our electrophysiological data shows that exogenous GAL in the DMH did not alter the activity of RVM On- and Off-cells that are non-serotonergic pain control neurones. Therefore, we propose that the RVM cells expressing c-Fos following exogenous GAL treatment may have been RVM Neutral-cells, a subpopulation of which are serotonergic [63] and which were not studied in the present electrophysiological experiment. The fact that the DMH GAL-driven descending pronociceptive drive is independent of RVM On- and Off-like cell activity is very interesting in terms of pain management, since many centrally acting analgesic compounds (opioids, cannabinoids and non-steroidal anti-inflammatory drugs) reduce pain by increasing the discharge rate of antinociceptive RVM Off-cells and/or by inhibiting the discharge rate of pronociceptive RVM On-cells [61].

In SHAM animals, repetitive limb extension alone or exogenous GAL administration alone in the DMH activated the descending PAG-RVM-spinal cord pathway as revealed by c-Fos expression. However, application of exogenous GAL simultaneously with repetitive extension of the limb failed to increase c-Fos expression in the PAG-RVM circuitry of SHAM animals, suggesting that together the two stimulation procedures counteracted each other's effect, leading to a general inhibition of this circuitry. The increased c-Fos expression in the RVM by the pronociceptive exogenous GAL treatment alone might reflect activation of RVM serotonergic cells. While the serotonergic system has a complex role in pain control, there is evidence suggesting that the net effect induced by RVM serotonergic neurones is facilitation of nociception [64]. It should be noted here that serotonergic RVM neurones are not On- or Off- cells [63] that were studied in the present electrophysiological experiment using noxious heat and shown not to be influenced by exogenous GAL. We propose that the GAL-induced descending action may have induced activation of medullo-spinal serotonergic neurones shown as increased c-Fos expression in the RVM and resulting in the relay of pronociceptive action to the spinal cord.

The increased expression of c-Fos in the serotonergic DRN after limb extensions is in line with a role of this nucleus in ascending [65] and descending [66] pain modulatory pathways. Similarly, increased expression of c-Fos of DRN after exogenous GAL in the DMH is not unexpected as the DMH projects directly to the DRN [67] and the activity of DRN serotonergic neurones is influenced by GALR1 present on their soma and proximal dendrites [68]. It still remains to be studied through which mechanisms the DRN might be involved in the relay of the descending pronociceptive effect driven by exogenous GAL in the DMH.

### 4. Activation of the noradrenergic LC by exogenous GAL in the DMH and noxious peripheral stimulation varies between SHAM and ARTH animals

Previous studies have demonstrated that the LC responds to noxious stimulation, as revealed e.g. by c-Fos expression [69], while it is a major source of spinal noradrenaline and descending noradrenergic control of nociception [70], [71]. In the present study, exogenous GAL treatment of DMH alone failed to influence c-Fos expression of LC in SHAM or ARTH animals. However, following repetitive limb extensions, c-Fos expression of LC was increased in SHAM but not ARTH animals. Interestingly, the peripheral stimulation-induced increase of c-Fos expression in the LC was predominantly ipsilateral, while ascending nociceptive signals activate the LC contra- or bilaterally [71]. A potential explanation for the ipsilaterally increased c-Fos expression after peripheral stimulation in the present study is that it reflected activation of descending pain modulation pathways descending predominantly ipsilaterally rather than processing of the ascending afferent volley that is expected to be contra- or bilateral. The DMH has a strong galaninergic output to various brain areas [72], including the LC [65], and GAL has been shown to decrease neuronal firing in LC [73]. While these findings suggest that the DMH may directly modulate activity of the LC, they still leave open what is the underlying mechanism and functional significance of the finding that exogenous GAL treatment of the DMH together with repetitive limb extensions increased c-Fos activity in the LC of ARTH but not SHAM animals.

### 5. Influence of arthritis and repetitive limb movement

The increase of c-Fos expression in the VLPAG and to a lesser extent in the RVM of SAL-treated ARTH animals indicates an overall increase in the tonic activity of the PAG-RVM-spinal cord pathways after the induction of experimental monoarthritis, which is in accordance with the enhancement of descending inhibitory circuits during chronic inflammation [74]–[77]. Interestingly, limb extension in the ARTH group decreased c-Fos expression in the VLPAG suggesting that acute noxious mechanical stimulation of the injured knee dampens tonic descending inhibition mediated by the VLPAG. On the other hand, the increase in c-Fos expression in the RVM, taking into account that the RVM can either facilitate or inhibit nociception [78], could indicate that this nucleus is engaged in descending facilitation during acute noxious stimulation of ARTH animals, as shown for other chronic pain disorders [79]–[81].

Our electrophysiological results showed that before any drug treatments both the baseline and the peripheral stimulus-evoked response in antinociceptive RVM Off-like cells were lower in ARTH than SHAM animals, while there was no difference in the pre-treatment heat-evoked activity of pronociceptive RVM On-like neurones of ARTH and SHAM animals. This finding suggests that a decreased activity of RVM Off-like cells contributes to hyperalgesia in ARTH animals. However, it does not exclude the possibility that among descending facilitatory mechanisms contributing to hyperalgesia in ARTH animals were other cell types of the RVM, in particular medullospinal serotonergic neurones, or other brainstem nuclei.

Concerning the DRN, the expression of c-Fos after repetitive limb extensions was increased, when compared with SAL-treated ARTH animals, but similar to the expression in c-Fos in SHAM after limb extensions, suggesting that the nociceptive processing through this pathway is not enhanced after the induction of experimental monoarthritis. By contrast, it is possible that the noxious stimulation-evoked activation of the LC is impaired in ARTH animals, since c-Fos expression was decreased when compared to SHAM animals after limb extensions and unaltered when compared to SAL-treated ARTH animals.

Without noxious stimulation, exogenous GAL in the DMH of ARTH animals appeared to dampen tonic descending inhibition (as indicated by decreased c-Fos expression in the VLPAG) while it enhanced the tonic activity of pronociceptive serotonergic (indicated by increased c-Fos expression in the DRN and RVM), but not noradrenergic (unaltered c-Fos expression in the LC) circuits. However, when combined with limb extensions, both tonic descending inhibition (decreased c-Fos expression in the VLPAG) and the activity of pronociceptive serotonergic areas (decreased c-Fos expression in the RVM and DRN) were diminished. This finding indicates that in ARTH animals, exogenous GAL in the DMH exerts differential effects under basal and noxious stimulation-evoked conditions. A differential effect has also been reported while studying the role of GAL in the presence/absence of stress [49].

By contrast, only the combination of exogenous GAL injection in the DMH and limb stimulation was able to enhance the activity of the noradrenergic pain system as evidenced by the strong increase of c-Fos expression in the LC. Although noradrenergic pathways were up to recently considered to exert mostly inhibitory influences on spinal nociception, Hickey and collegues [59] recently demonstrated that a specific subpopulation of LC neurones enhances the processing of nociceptive information and could thus partly contribute to behavioural hyperalgesia in chronic inflammation. Further studies are needed to find out whether LC is involved in mediating the descending pronociceptive effect elicited by exogenous GAL in the DMH.

## Conclusions

In the present study, we demonstrate a pronociceptive GALR1-mediated role for hypothalamic GAL in experimental monoarthritis. Exogenous GAL in the DMH appeared to exert differential effects upon the brainstem pain modulatory areas; the effect varied between the experimental group (healthy or arthritic animals), brainstem nucleus (PAG, RVM, DRN, or LC), and the presence or absence of concomitant noxious stimulation. Finally, the results suggest that further studies evaluating the potential applicability of GALR1 antagonists in the control of chronic inflammatory pain are needed.
